# Case report: Block recession calcaneoplasty of the calcaneal tuber for treating lateral superficial digital flexor tendon luxation in a dog

**DOI:** 10.3389/fvets.2022.969414

**Published:** 2022-12-13

**Authors:** Sanghyun Nam, Haebeom Lee, Yoonho Roh, AhRan Kang, Daehyun Kim, Seongmok Jeong, Jaemin Jeong

**Affiliations:** ^1^College of Veterinary Medicine, Chungnam National University, Daejeon, South Korea; ^2^Division of Small Animal Surgery, Department of Clinical Veterinary Medicine, Vetsuisse Faculty, University of Bern, Bern, Switzerland

**Keywords:** superficial digital flexor tendon, block recession calcaneoplasty, temporary restraining pin, retinaculum repair, dog

## Abstract

A 4-year-old, intact, female, Collie was presented with 5 month history of right hindlimb lameness. Lateral luxation of the superficial digital flexor tendon (SDFT) was diagnosed on the basis of the clinical, radiographic and ultrasonographic finding. Intraoperatively, shallow right calcaneal tuber was observed. Block recession calcaneoplasty with retinaculum repair using anchor screw were performed to manage SDFT luxation. Additionally, temporary restraining pin was placed on lateral aspect of the calcaneal tuber. The patient demonstrated mild lameness at 2 weeks postoperatively and improved to normal limb function at 12 weeks postoperatively. As the gold standard of surgical techniques for SDFT luxation has not yet been reported, block recession calcaneooplasty may be an alternative surgical option for patients with calcaneal morphologic abnormalities causing SDFT luxation.

## 1. Introduction

Superficial digital flexor tendon (SDFT) luxation from the calcaneal tuber is a relatively rare condition that causes hindlimb lameness in dogs. The pathophysiology of SDFT luxation remains unclear, although some predisposing factors have been reported including evident calcaneal morphologic abnormalities such as a flattened, sloped, or convex surface of the calcaneal tuber tip and breed predispostion. These morphologic abnormalities might lead to medial or lateral SDFT luxation, with the latter being reported to be more common (1–4).

Previous studies have recommended surgical repair for SDFT luxation through reconstructing the damaged retinaculum with a suture anchor screw (5). Although retinacular reconstruction with augmentation using temporary restraining pin placement has been reported to have a favorable prognosis, information on the management of SDFT luxation is limited (5). A flattened calcaneal morphologic abnormality is considered a predisposing factor for SDFT luxation. Although a recent study reported a successful long-term outcome through abrasion calcaneoplasty in 12 dogs, there is still a lack of studies related to surgical description and clinical outcomes of calcaneal abnormality correction in veterinary medicine (6).

This case report describes the successful surgical outcomes of block recession calcaneoplasty with reconstruction of the ruptured retinaculum using a suture anchor and placement of a temporary restraining pin for management of traumatic lateral SDFT luxation in a dog with flattened calcaneal tuber.

## 2. Case description

**Figure 1:**
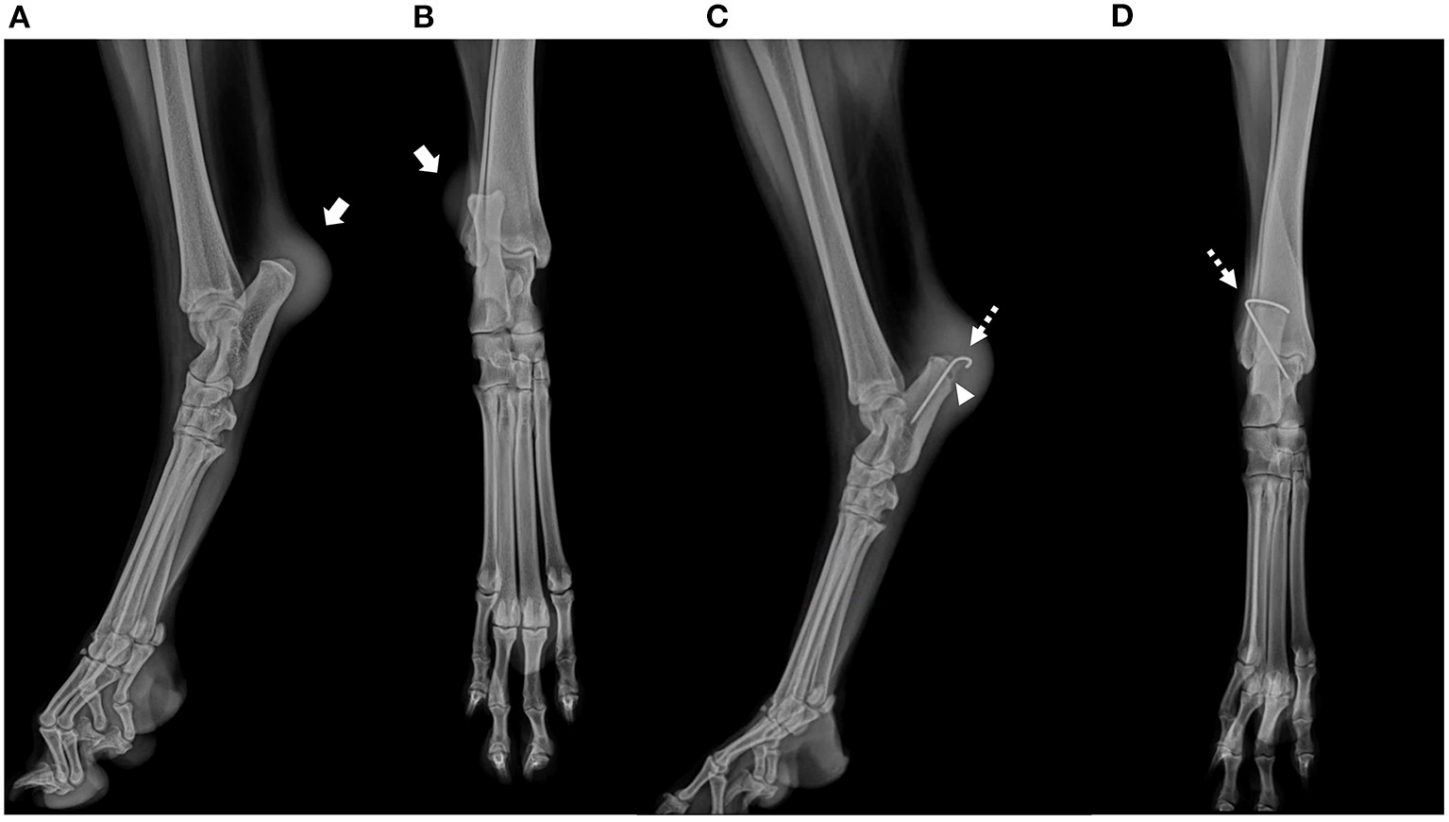
Preoperative mediolateral **(A)** and craniocaudal **(B)** radiographs of the affected right calcaneal region. Note soft tissue swelling in the cauda-ventral aspect of the calcaneal tuber of the right tarsal joint, in absence of bone lesion. (arrow). Postoperative mediolateral **(C)** and craniocaudal **(D)** radiographs. Temporary restraining pin placed on the lateral aspect of calcaneal tuber **(C,D, dotted arrow)**. A 2.4 × 8.5 mm biocomposite suture anchor (SutureTak; Arthrex Inc. Naples, FL) placed on medial aspect of calcaneal tuber **(C, arrow head)**.

A 4-year-old, 37.5 kg, intact, female, Collie was referred to Chungnam National University Veterinary Medicine Teaching Hospital with soft tissue swelling around right calcaneus tuber and 5-month history of non-weight-bearing lameness of the right hindlimb after traumatic event of slipping on the floor. On orthopedic examination, a pain reaction was observed during manipulation of the right tarsal joint, and the SDFT was luxated laterally during tarsal joint flexion. No orthopedic abnormalities were observed in the contralateral tarsal joint of the right hindlimb. Radiographs revealed soft tissue swelling in the cauda-ventral aspect of the calcaneal tuber, in absence of bone lesion (Figure 1). In the ultrasound examination, a mixed hypoechoic and anechoic lesion was observed at the proximal level of the calcaneal tuber.

The dog was diagnosed with traumatic right hindlimb lateral SDFT luxation and surgical management of luxated SDFT was offered to client. The dog was premedicated with midazolam 0.2 mg/kg i.v. (Midazolam; Bukwang Pharm Co., Ltd. South Korea). After induction with propofol 6 mg/kg i.v. (Anepol; Hana Pharm Co., Ltd. South Korea), general anesthesia was maintained with isoflurane (Isoflurane; Hana Pharm CO., Ltd. South Korea) and oxygen provided by a rebreathing circuit system through endotracheal intubation. Remifentanil 0.1–0.3 μg/kg/min i.v. (Remiva; Hana Pharm Co., Ltd. South Korea) was administered for analgesia. Cefazolin 22 mg/kg i.v. q90 min (Cefazoline; Chong Kun Dang. South Korea) was administered 30 min before incision and repeated every 90 min. The dog was placed in left lateral recumbency with the right hindlimb placed uppermost. The skin was aseptically prepared for surgery.

**Figure 2:**
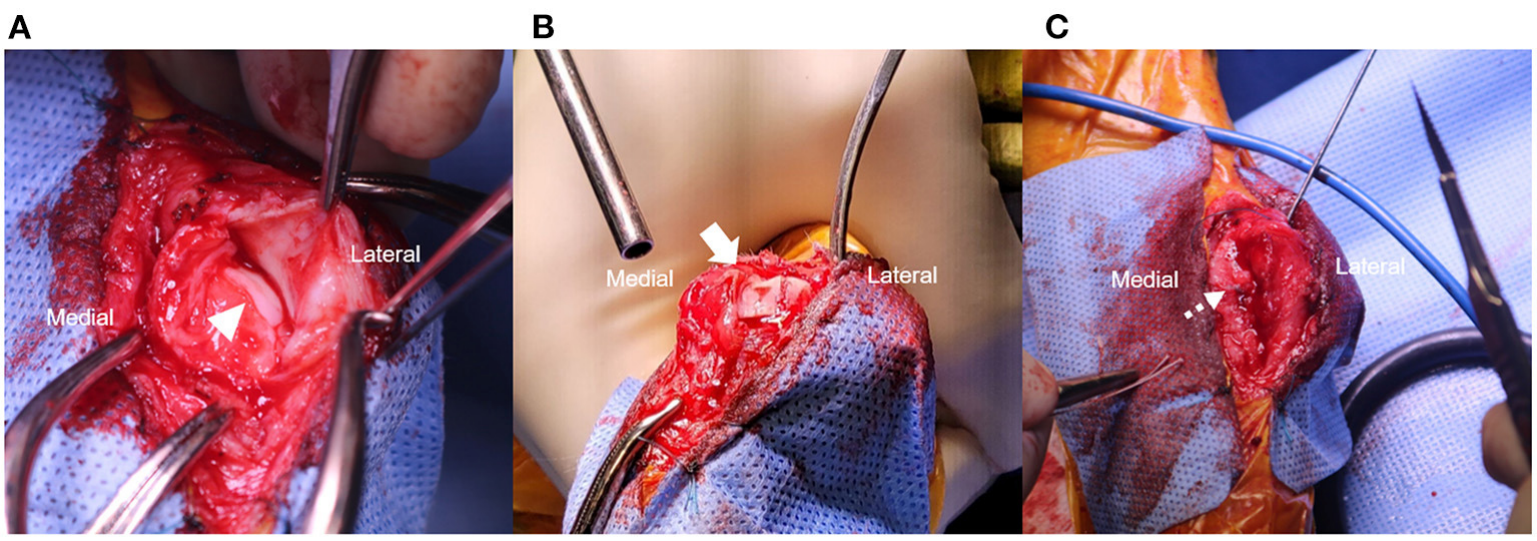
Intraoperative photographs of a flattened groove of calcaneal tuber [**(A)**, arrowhead]. Note the deepened depth of the calcaneal tuber following calcaneoplasty [**(B)**, arrow]. Medial retinaculum was repaired using 2.4 × 8.5 mm biocomposite suture anchor (SutureTak; Arthrex Inc. Naples, FL) and 2-0 non-absorbable braided suture (FiberWire; Arthrex Inc. Naples, FL) [**(C)**, dotted arrow]. Temporary restraining pin was placed on the lateral aspect of calcaneal tuber.

**Figure 3:**
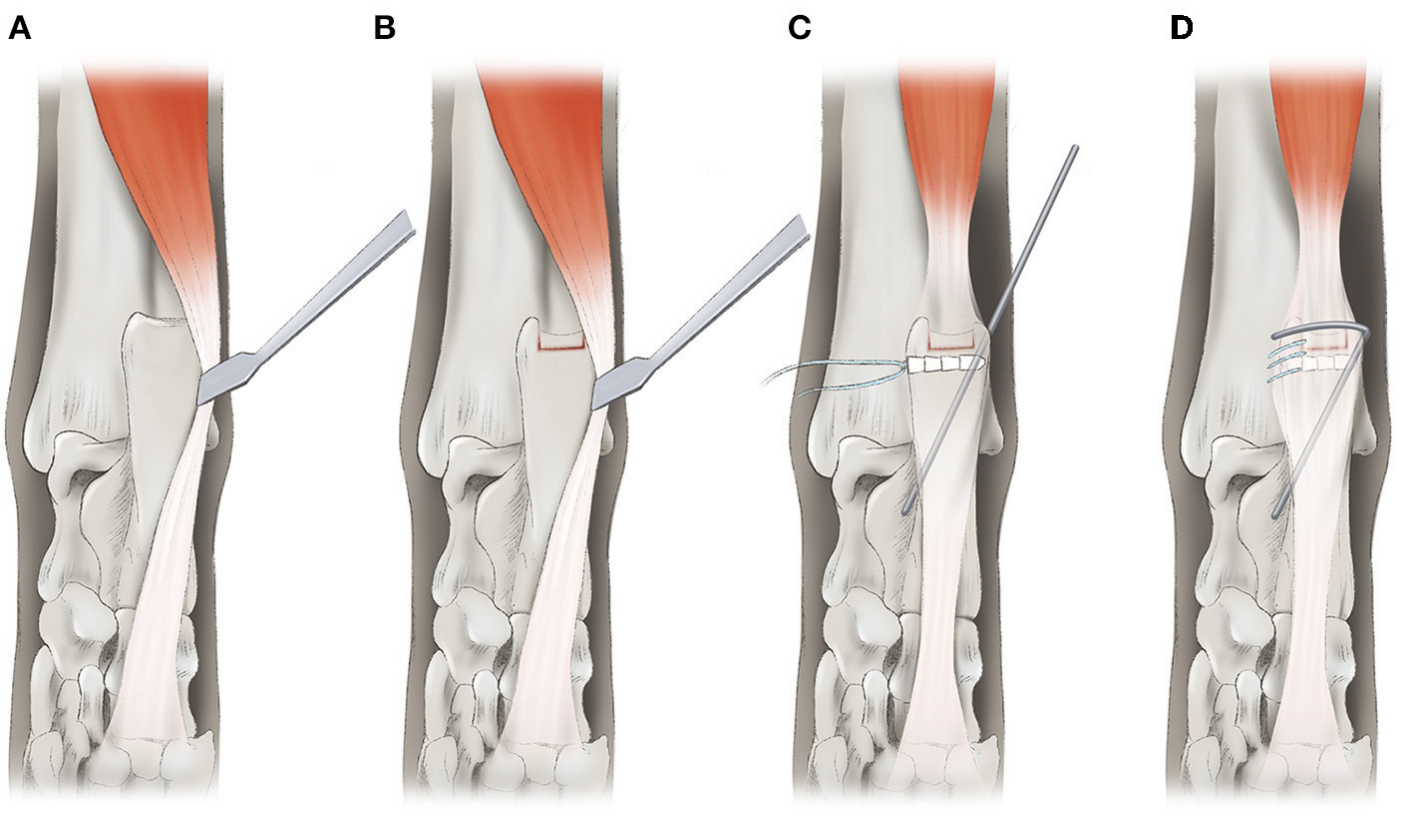
A schematic diagram of block recession calcaneoplasty **(A,B)**. The bone flap was made by using a hobby saw and mini lambotte osteotome **(B)**. Deepening of the calcaneal tuber was performed using a high-speed burr and the bone flap was replaced on the cancellous bone bed. The retinaculum was repaired using a 2.4 × 8.5 mm biocomposite suture anchor (SutureTak; Arthrex Inc. Naples, FL) and 2-0 non-absorbable braided suture (Fiberwire; Arthrex Inc. Naples, FL) in simple continuous pattern **(C,D)**. A temporary restraining pin was placed on the lateral aspect of calcaneal tuber to prevent reluxation of SDFT tendon.

A medial skin incision was made along the right calcaneus. The medial bursa was incised, and the SDFT was retracted laterally with a Hohmann retractor to expose the calcaneal tuber (Figures 2, 3). A flattened and smooth surface of the calcaneal tuber was identified on gross observation (Figure 2A). A block recession calcaneoplasty was performed (length × width × depth: 8.5 × 4 × 3 mm) in order to deepen the groove (Figures 2B, 3B). The width of the groove block was determined with width of the SDFT. The block recession was performed using hobby saw and mini lambotte osteotome. The bone flap was made and the underlying cancellous bone was deepened with 1- and 3-mm round ball type high speed burr. The depth of calcaneal tuber groove was deepened by 1 mm at each time to avoid tendon locking while evaluating the gliding function of the SDFT. The bed of the block was deepened to 3 mm until the SDFT glided smoothly without impingement (Figure 2B).

The ruptured medial retinaculum was repaired using a 2-0 non-absorbable braided suture (FiberWire; Arthrex Inc. Naples, FL) by a simple continuous suture pattern and a 2.4 × 8.5 mm biocomposite suture anchor (SutureTak; Arthrex Inc. Naples, FL) (Figures 2C, 3C,D). The repair was augmented by placing a bicortical temporary restraining pin (1.2-mm K-wire) on the lateral aspect of the calcaneal tuber, proximo-distally and latero-medially. The restraining pin was bent medially for covering SDFT to prevent lateral luxation (Figure 3D). The surgical site was lavaged with sterile saline, and swabbed for culture and sensitivity tests. The subcutaneous tissue and skin was closed routinely.

Postoperative radiographs were taken immediately to confirm the position of the temporary restraining k-wire (Figure 1). Remifentanil (Remiva; Hana Pharm CO., Ltd. South Korea) was continued for analgesia for 24 h postoperatively at 0.1–0.3 μg/kg/min with constant rate infusion. Additional analgesia and the anti-inflammatory drug meloxicam (Metacam injection, Boehringer Ingelheim) was administered, 0.1 mg/kg s.c. q24 h for 5 days was administered 1 h postoperatively. Amoxicillin clavulanate (12.5 mg/kg, q12h) and clindamycin (11 mg/kg, q24h) were administered as postoperative antibiotics for 3 days due to chronic inflammation of the surgical site and concerns about using non-absorbable braided suture. A modified Robert Jones bandage was applied for the first 24 h postoperatively. Surgical wound healing was uneventful without bacterial culture growth.

**Figure 4:**
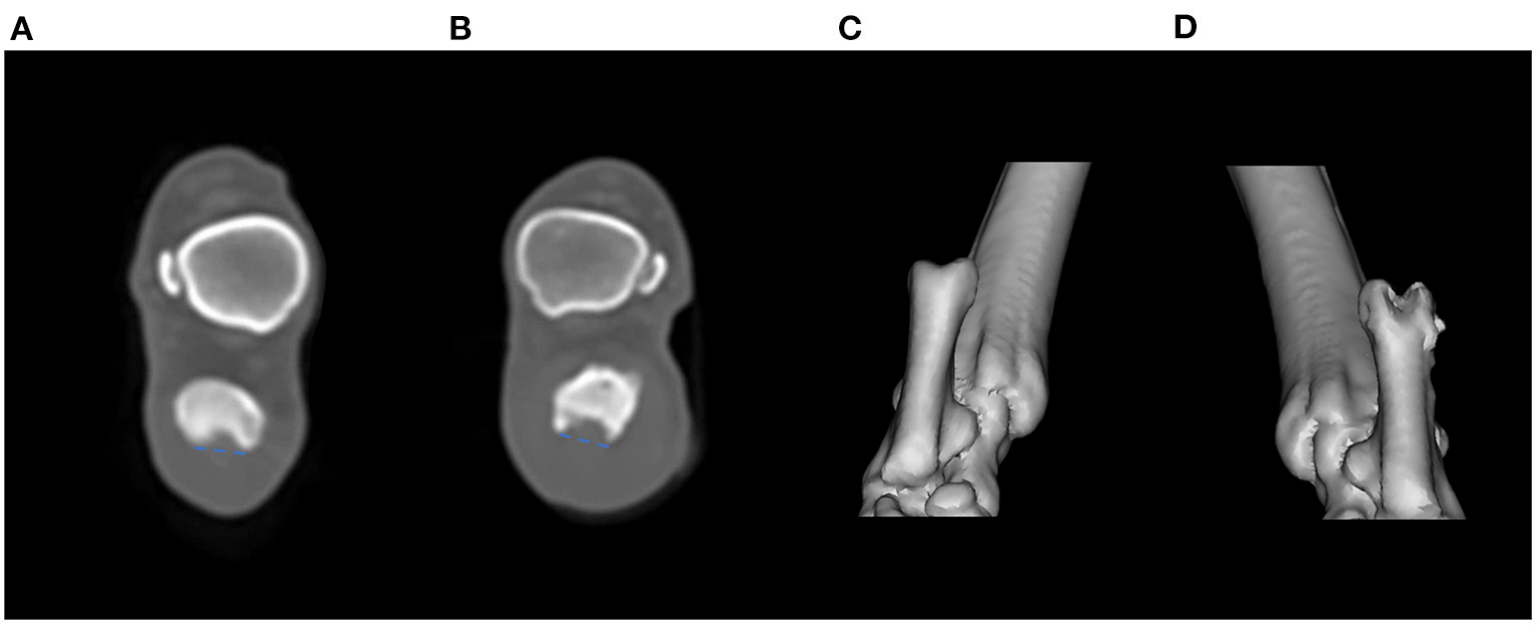
Postoperative computed tomography of the calcaneal tuber **(A,B)**. The calcaneal groove depth was measured from the level of medial and lateral calcaneal tuber to the calcaneal groove. The depth of the unaffected left calcaneal tuber **(A)** was 2 mm, and that of the right was 3 mm postoperatively **(B)**. Caudocranial view of the three-dimensional reconstruction of the bilateral calcaneal tuber [**(C)**: left, **(D)**: right]. Note that the right calcaneal tuber groove is deepened compared with the left side.

The patient was discharged 8 days postoperatively. Immobilization was done for 6 weeks postoperatively, and after 6 weeks the owner was educated regarding leash walking with a supporting sling harness for 2 weeks. Computed tomography (CT) was performed 7 weeks postoperatively after removing the temporary restraining k-wire, confirming that the groove depth of the right calcaneal tuber was deeper than the unaffected contralateral groove (right, 3 mm; left, 2 mm) (Figure 4). The limb function was evaluated using 5-point visual lameness scoring system in each follow-up examination (16). At 6 weeks postoperatively, the patient showed mild weight bearing lameness (lameness score; 1/4) and gradually improved (lameness score; 0/4) until 12 weeks postoperatively. The patient was examined until 14 months postoperatively; no signs of pain or lameness were noted.

## 3. Discussion

SDFT luxation has been previously reported in a few studies; however, its etiology remains unclear in veterinary medicine (1, 5). In this report, the SDFT luxation with a flattened and convex surface of calcaneal tuber was successfully managed with block recession calcaneoplasty to deepen the groove and reconstruction of the ruptured medial retinaculum by a 2-0 fiber wire and suture anchor. To the best of author's knowledge, this is the first case report of SDFT luxation treated with block recession calcaneoplasty. The patient showed normal limb function without lameness at 12 weeks postoperatively and maintained until 14-month follow-up.

Abnormal morphology of calcaneus is considered one of the major factor causing SDFT luxation (5, 7). According to a previous study, deformity that could increase the strain of the retaining soft tissue includes abnormal form of the calcaneus causing bone to bow in sagittal, axial, or frontal planes, although such defect should be evident radiographically. However, deformity such as an underdeveloped medial or lateral process would be less likely to be detected radiographically (Figure 1). Thus, preoperative 3-dimentional reconstruction can be helpful for surgical decision making by pre-measuring the groove depth and width of the contralateral groove. Previous surgical treatment did not focus on resolving underlying anatomical deformity, thus performing a block recession calcaneoplasty to deepen the groove between the medial and lateral process of the calcaneal tuber would be plausible (5). In our case, clear evidence of bone deformity was not observed in the radiographs (Figures 1A,B), although an flattened calcaneal groove was observed intraoperatively (Figure 2A).

A shallow groove causing luxation is also observed in patellar luxation. The underlying cause of the patellar luxation is not entirely known, although one of the elements known to cause patellar luxation is a shallow calcaneal groove with poorly developed lateral and medial ridges (8, 9). Block recession is one of the trochleoplasty techniques for fixing the anatomical abnormality of the trochlear groove (10, 11). In human medicine, peroneal tendon subluxation has been reported to be caused by shallow, flat, convex anatomical morphologies of the peroneal groove. Surgical repair of the peroneal tendon subluxation involves deepening the peroneal groove by removing the corticocancellous bone window in the posterior fibula, deepening the groove, and placing the cortical bone on the groove. Posterior fibular groove deepening for peroneal tendon subluxation had good outcomes, and activity returned to normal 3 months postoperatively (12–15). Previous studies report surgical management methods of SDFT luxation either through retinaculum reconstruction using a suture anchor and temporary restraining pin or abrasion calcaneoplasty (5, 6). Several complications including fracture due to weakening of the calcanei and re-luxation of the SDFT or soft tissue complication due to inadequate external coaptation have been reported regarding to surgical management of SDFT luxation (2, 5). The block recession calcaneoplasty in this case was successful in managing the SDFT luxation in the long-term follow-up without any complications.

A recent retrospective study from United State, New Jersey, using abrasion calcaneoplasty and primary retinaculum repair in dogs to treat superficial digital flexor tendon luxation was reported coincidentally and in parallel to ours (6). They reported long-term outcomes of resolution of clinical lameness in all 12 dogs (6). As they did abrasion calcaneoplasty and primary retinaculum repair to address SDFT luxation, our case performed block recession to deepen the groove of calcaneus, suture anchors (SuterTak; Arthrex Inc/ Naples, FL) to repair the retinaculum and kirschner wire to temporarily restrain the SDFT luxation.

The placement of a temporary restraining pin was successful in protecting the surgical repair in our case and did not exhibit any signs of implant loosening, infections, and SDFT re-luxation. The temporary restraining pin was removed at 7 weeks postoperatively. Re-luxation of SDFT has been reported in 2 (9%) out of 23 cases surgically treated after removing the temporary restraining pin in 1 clinical series (5), although no sign of SDFT re-luxation after removing the temporary restraining pin was observed in our case. The reported risk factors of re-luxation of SDFT include inadequate or poor quality of lateral restraining tissue relative to the size of the patient (5). Postoperative immobilization of the tarsal joint for 4–8 weeks was recommended in some studies (2, 8), while in our case, 6 weeks of postoperative immobilization was done.

This case report had limitations. First, we only reported a single case. Additionally, we did not evaluate the biomechanical effect of deepening the calcaneal groove and it's width on the SDFT, which in further studies may be investigated. Finally, evaluation of the 3-dimensional reconstructed calcaneal morphology was not done preoperatively and we did not compare with the contralateral side in this dog nor with normal unaffected dogs. In future studies, comparing the calcaneal groove and shape morphologies of normal and SDFT luxation cases would help to explore the etiology of SDFT luxation.

## 4. Conclusion

In conclusion, lateral SDFT luxation was successfully managed by block recession calcaneoplasty with suture anchor and temporary restraining pin. No recurrence of SDFT luxation was observed after removing the temporary restraining pin. Block recession calcaneoplasty can be considered an alternative surgical option to manage SDFT luxation caused by a shallow calcaneal groove. Further study is needed to evaluate the effect of the depth and the shape of the calcaneal tuber groove to the SDFT luxation.

## Data availability statement

The raw data supporting the conclusions of this article will be made available by the authors, without undue reservation.

## Ethics statement

Ethical review and approval was not required for the animal study because this study is a case report of examinations and surgery performed for the purpose of treatment of patients, and no action contrary to treatment was performed. Written informed consent was obtained from the owners for the participation of their animals in this study.

## Author contributions

SN and AK performed clinical management of the case, and wrote and edited the manuscript. HL performed the surgery. JJ and HL contributed to the conception of the case report and revised the manuscript. JJ, DK, and SJ supervised the clinical management of the case. All authors contributed to preparation and final approval of the manuscript.
